# Supplementary material for: The Impact of Glucose-Based or Lipid-Based Total Parenteral Nutrition on the Free Fatty Acids Profile in Critically Ill Patients
Source: Nutrients. 2020 May 11;12(5):1373. doi: 10.3390/nu12051373 (PMC7284730; doi:10.3390/nu12051373)
Supplement: Supplementary file 1 [file nutrients-12-01373-s001.pdf]

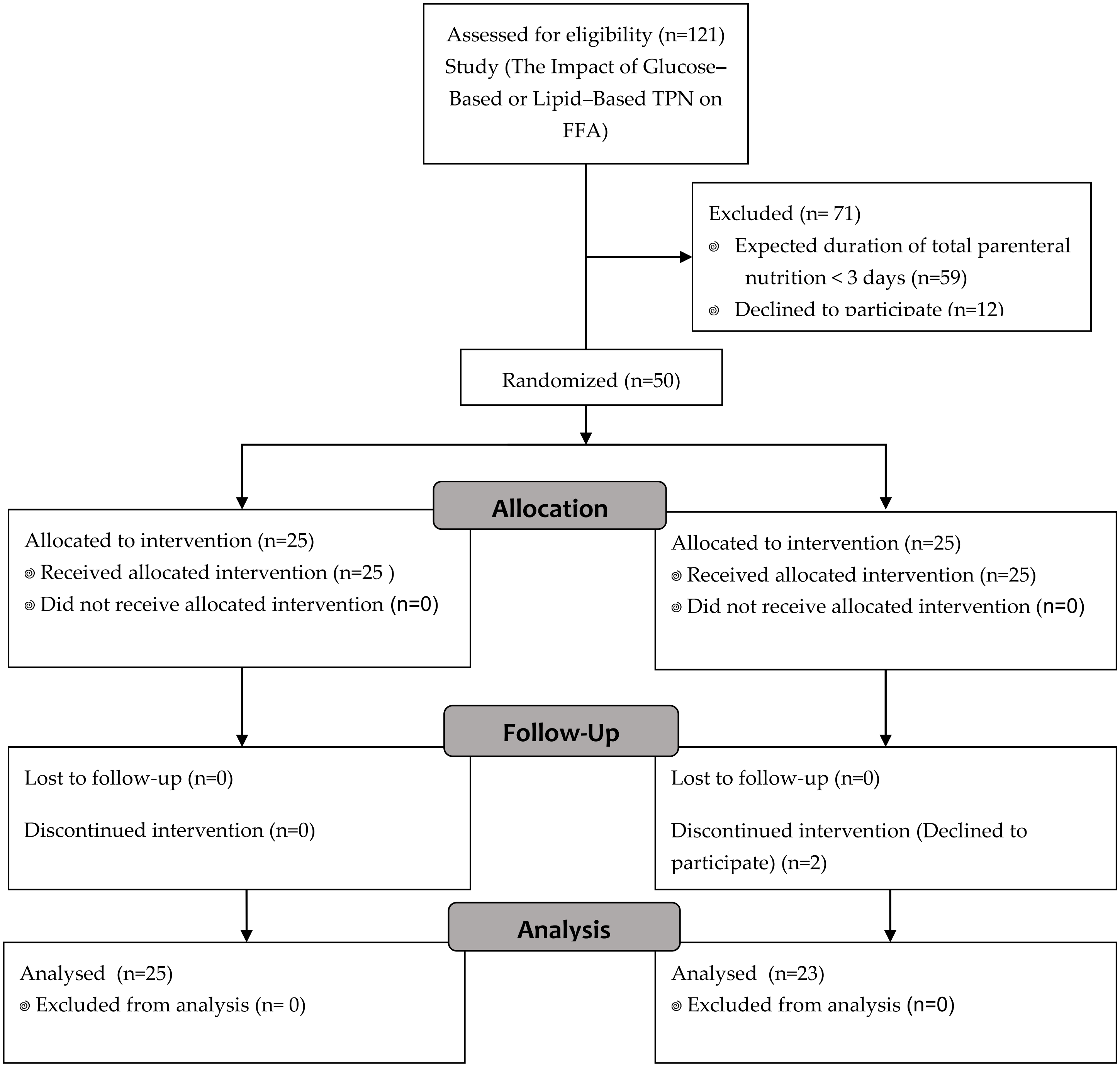

Figure S1. Flow diagram for patients enrolled in the study "The Impact of Glucose–Based or Lipid–Based Total Parenteral Nutrition on the Free Fatty Acids Profile in Critically Ill Patient"

Table S1. Plasma concentrations of selected hormones of the adipose tissue (Mean values with standard deviations).

Variable	Group	Day 0	Day 1	Day 3	Day 6	Day 9	Day 14	Day 28	*p*-Value
Resistin	GG	26.94±7.40	24.36±9.67	24.58±10.08	22.08±10.80	23.56±10.65	24.02±11.42	26.02±8.03	n.s.
(ng·mL^{-1})	LG	20.53±12.27	22.78±11.05	19.42±11.62	21.30±12.13	18.35±11.74	16.76±11.46	23.28±5.31	n.s.
Leptin	GG	4.04±3.48	14.32±13.69	16.69±14.66	14.34±12.15	16.24±12.41	14.05±11.01	14.23±9.32	n.s.
(pg·mL^{-1}l)	LG	6.77±3.66	8.59±6.22	6.95±3.97	9.13±6.27	5.88±4.24	3.92±4.21	3.92±3.21	n.s.
PAI–1	GG	62.84±38.05	65.22±41.11	57.85±33.86	54.19±32.99	88.78±47.46	68.99±53,40	81.99±48.63	n.s.
(ng·mL^{-1})	LG	52.91±28.06	40.91±26.03	41.48±28.76	47.07±22.12	54.65±32.51	53.15±30.53	40.46±17.01	n.s.

GG = Group G, glucose based total parenteral nutrition; LG = Group L, lipid based total parenteral; PAI–1, plasminogen activator inhibitor-1; n.s., non–significant
